# Extremely flat metal films implemented by surface roughness transfer for flexible electronics†

**DOI:** 10.1039/c8ra00298c

**Published:** 2018-03-19

**Authors:** Kisoo Kim, Sungjoo Kim, Gwan Ho Jung, Ilhwan Lee, Sungjun Kim, Juyoung Ham, Wan Jae Dong, Kihyon Hong, Jong-Lam Lee

**Affiliations:** Division of Advanced Materials Science, Department of Materials Science and Engineering, Pohang University of Science and Technology (POSTECH) Pohang 790-784 Korea jllee@postech.ac.kr

## Abstract

We present an innovative approach to fabricate an extremely flat (EF) metal film which was done by depositing metal on an extremely flat mother substrate, then detaching the metal from the substrate. The detached flexible metal films had a roughness that was within 2% of the roughness of the mother substrate, so EFs with *R*_a_ < 1 nm could be fabricated using the surface roughness transfer method. With quantitative analysis using *in situ* synchrotron XPS, it was concluded that the chemical reaction of oxygen atoms with the metal film played a critical role in designing a peel-off system to get extremely flat metal films from the mother substrate. The OLED was successfully implemented on the metal film. The OLED's luminance could be increased from 15 142 to 17 100 cd m^−2^ at 25 mA m^−2^ by replacing the glass substrate with an EF copper (Cu) substrate, due to the enhanced heat dissipation during the operation. This novel method can be very useful for mass production of large scale, low-cost and high quality metal films using roll-to-roll process.

## Abstract 2

We present an innovative approach to fabricate an extremely flat (EF) metal film which was done by depositing metal on an extremely flat mother substrate, then detaching the metal from the substrate.

## Graphical abstract

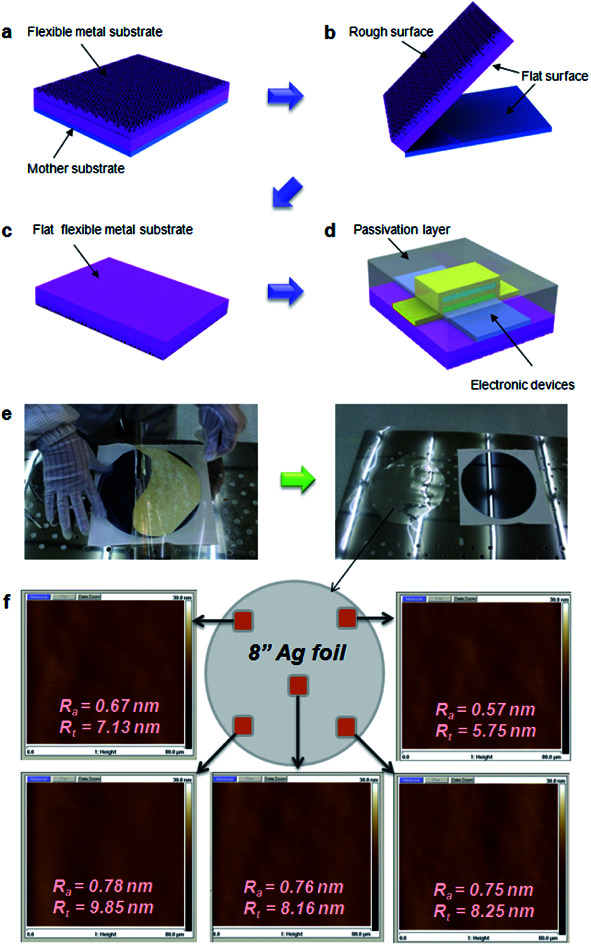

## Introduction

Flexible electronics are pliable, thin, light-weight, impact resistant, and adaptable in design, so they have many possible applications in mobile information displays.^1–6^ Therefore, to enable flexible electronics, flexible substrates (plastic, steel) and fabrication methods have been studied in numerous reports.^7–12^ However, the required flexible nature of the substrate causes the following fundamental challenges; polymeric substrates have limitations of process temperature (<350 °C), at which thin film transistors with high carrier mobility and stability cannot be fabricated.^13–15^ The difference in coefficient of thermal expansion (CTE) between plastic and silicon-based materials causes cracks in devices during the heating processes.^14^ To overcome such problems, a substrate transfer technology using laser ablation has been introduced.^16–19^ But, the laser lift-off technologies are not appropriate for organic electronics because laser irradiation of the a-Si : H sacrificial layer does not allow the electronic devices to be completely separated from the glass substrate; instead, an additional physical peel-off process is needed.^17,20^ This physical peel-off process damages the organic active layer on top. A polyimide sacrificial layer also has been used to suppress the defects, but the thermal budget of polyimide films is low to obtain dielectric films with high quality.^19^ The high energy of the laser would also damage the surface of the metal layer.^18^ As an alternative approach, steel substrates have been studied, but they have rough surface, so additional planarization processes are required.^21–23^ A polishing process of the conventional steel substrate and a polymer coating process on the substrate do not satisfy the requirements for low price, low defect density, and low tack time.^24,25^ The steel substrate also has still high surface roughness (*R*_a_ > 2 nm) after the planarization.^26,27^ Use of a metal substrate is ideal in flexible electronics because it has excellent mechanical strength, and heat resistance greater than that of a glass substrate. However, most metal films have very rough surface to be used in the devices. So, a novel, low-cost, high-throughput, flexible and extremely flat metal films is needed.

Here, we present an innovative approach to fabricate an extremely flat (EF) metal films by depositing a metal film on an extremely flat mother substrate, then detaching the metal film from the substrate. The detached films had the roughness that was within 2% of the roughness of the mother substrate, so EFs with average roughness *R*_a_ < 1 nm could be fabricated without any polishing or planarization process. We demonstrated EF Ag film (8 inch, *R*_a_ = 0.57 nm, maximum roughness amplitude *R*_t_ = 5.75 nm) and Cu film (20 cm × 20 cm, *R*_a_ = 2.42 nm, *R*_t_ = 30.04 nm). Consequently, excellent flatness allows fabrication of various electronic devices on the detached metal surface. The optical and electrical performances of flexible organic light-emitting diodes (OLEDs) and organic photovoltaics (OPVs) using EF metal films were comparable to those on rigid glass substrate.

## Experimental section

### Preparation of EF metal film

Glass, indium-tin oxide (ITO) and silicon (single-side polished) were used as a mother substrate for fabricating EF metal film. The mother substrate was cleaned sequentially with acetone, isopropyl alcohol, and deionized water, on which metal films were deposited by various techniques such as a thermal evaporation, electron-beam evaporation, or electroplating. When the evaporation method was used, Ag films were deposited to 30 μm thickness at a rate of 1.0 nm s^−1^. The growth chamber pressure was maintained at ∼10^−8^ Torr during the deposition, and the substrate was held at room temperature. Then, adhesive support film was attached to the Ag film for the effective pee-off process in the large-area film. The support film with the 8 inch Ag film was successfully detached from the Si substrate. A large compressive stress could be built at the interface of the Ag film with the Si substrate due to their lattice parameter change (4.08 Å for Ag and 5.43 Å for Si). This led to easily peel-off of the Ag film from the Si substrate. Metal films (Cu, Ni or invar) could be grown on the mother substrates by evaporating to a thickness of several tens of nanometers. Subsequently, same kinds of metal films were electroplating to thicken the metal films to several tens of micrometers. To electroplate metal films on the mother substrate, the cathode (metal layers on mother substrate) and anode (bulk plating material stick) were electrically connected using a Keithley 2400 digital source meter to ensure constant current density. The temperature of the electroplating solution was maintained to be 60 °C. All films could be easily peeled off from the mother substrate.

### Fabrication of top-emission OLEDs

Glass (*R*_a_ = 1.3 nm) was used as the reference substrate. EF Cu (*R*_a_ = 1.4 nm) and rolled STS430 (*R*_a_ = 53.0 nm) substrate was used to test effects of surface roughness and top-emission OLEDs were fabricated on the substrates. The substrates were cleaned sequentially with acetone, iso-propyl alcohol and deionized water and then dried with a high-purity N_2_ gas. The substrates were transferred to a PECVD, then a 100 nm-thick SiO_2_ insulation layer was deposited on the substrate. Afterward, the insulated samples were transferred to a thermal evaporator. A metal (Ag 100 nm)/dielectric (WO_3_ 3 nm) structure was used for reflective anode electrodes. Then, WO_3_-doped 4,4′-*N*,*N*′-dicarbazole-biphenyl (CBP) as a 28 nm hole injection layer (HIL), intrinsic CBP as a 5 nm hole transport layer (HTL), Tris[2-phenylpyridinato-C2, N]iridium(iii) (Irppy_3_) doped CBP as a 20 nm emissive layer (EML), 1,3,5-tris(1-phenyl-1*H*-benzimidazol-2-yl)benzene (TPBi) as a 35 nm electron transporting layer (ETL), LiF as a 1 nm electron injection layer (EIL), Al (2 nm) and Ag (18 nm) as transparent cathodes, and WO_3_ (30 nm) as admittance matching layer were deposited in sequence. During deposition, the base pressure of the chamber was maintained as low as 10^−6^ Torr. The active area of the device was 30 mm × 30 mm.

### Characterization

X-ray photoelectron spectroscopy was carried out using synchrotron X-ray in 4D beam line in Pohang Accelerator Laboratory. The average surface roughness (at 5 randomly-selected points) of flexible EF metal films and mother substrates were measured by using a 3D-profiler (Wyko Veeco, NT1100) with a scan range of 1.2 mm × 0.9 mm and an AFM (Veeco-Digital instruments MMAFMLN-AM) in tapping mode using Sb-doped Si cantilevers. The current density–voltage (*J*–*V*) characteristics and luminance (*L*) of the OLEDs were measured with a Keithley 2400 source meter in a nitrogen ambient atmosphere. The *J*–*V* curves of OPVs were measured under air ambient with glass encapsulation using a Keithley 2400 source measurement unit. The photocurrent was measured under AM1.5G 100 mW cm^−2^ illumination from an Oriel 150 W solar simulator. The light intensity was determined using a mono-silicon detector calibrated by the National Renewable Energy Laboratory (NREL).

## Results and discussion

**Fig. 1:**
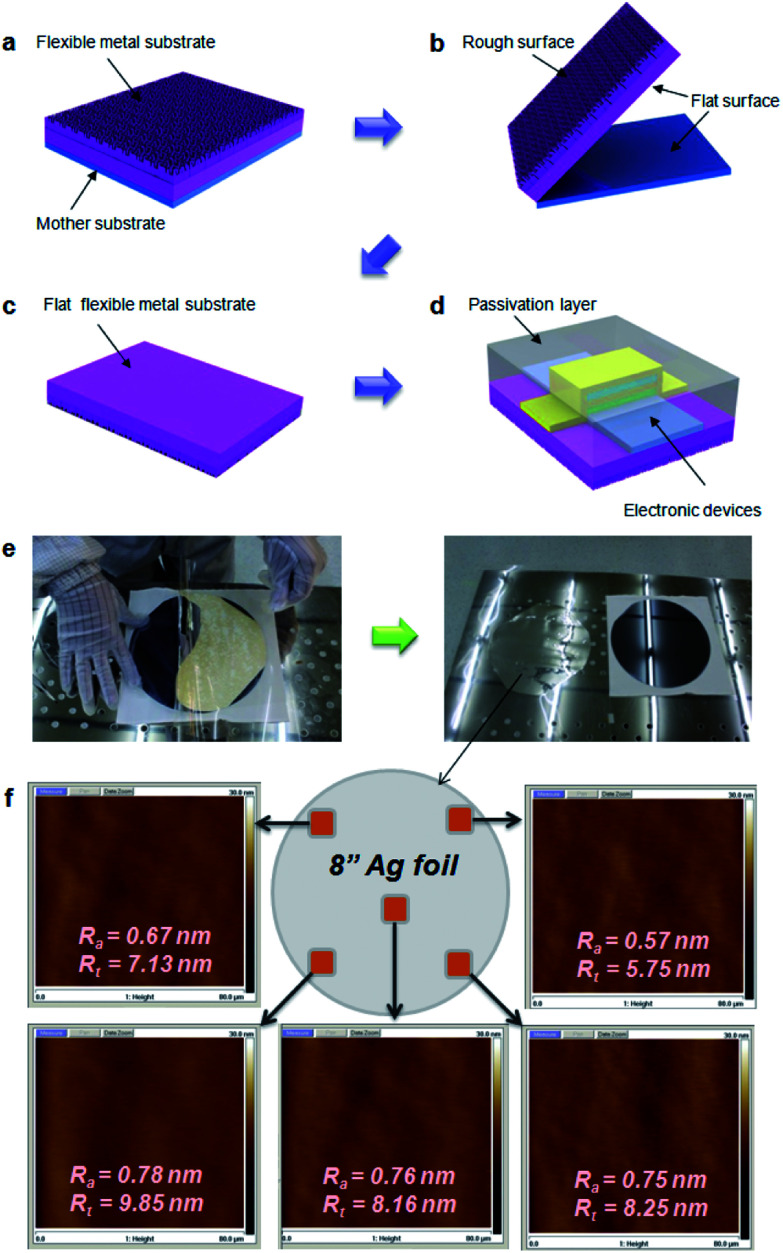
Generic process flow for extremely flat metal film using surface roughness transfer method. (a) Flexible metal film was formed on the mother substrate. (b) The flexible metal film was detached from the mother substrate. (c) The roughness of detached surface in the flexible metal film was transferred from the mother substrate. (d) Electronic devices were fabricated on top of detached surface in the metal film. (e) Photo images of peel-off process using Ag/Si wafer, corresponding (b) and (c) figures. (f) Surface roughness of an 8 inch Ag foil measured by AFM.

In order to fabricate EF metal films using a surface roughness transfer method, a flexible metal film was formed on mother substrate using various techniques such as thermal evaporation, electron-beam evaporation, or electroplating (Fig. 1a). In this study, we used Si wafers (8 inch), glass, ITO-coated glass (20 cm × 20 cm), and stainless steel (STS) sheets as the mother substrates. The flexible metal films with 30–100 μm-thick Ag, invar, Cu, or Ni were separately detached from the mother substrate (Fig. 1b). This allows the roughness of mother substrate to transfer to the detached surface of the flexible metals film, so EF metal films could be made by using the extremely flat mother substrates (Fig. 1c). Finally, various kinds of electronic devices such as OLEDs and OPVs can be fabricated on top of the detached surface of the metal films (Fig. 1d). As an example, EF Ag film was obtained by depositing a 30 μm-thick Ag film on an 8-inch Si wafer, then peeled-off from the Si wafer (Fig. 1e). The Ag film could be transferred to support film without any cracks (Fig. 1f) because of intrinsic flexibility of the Ag. The detached side of Ag film had 0.57 nm ≤ *R*_a_ ≤ 0.78 nm and 5.75 nm ≤ *R*_t_ ≤ 9.85 nm. X-ray photo-emission spectroscopy revealed that there is no transfer of atomic components by the peel-off process (Fig. S1†). In addition to Ag film, it has been confirmed that a large-area Cu film can be produced by using this method (Fig. S2†).

**Fig. 2:**
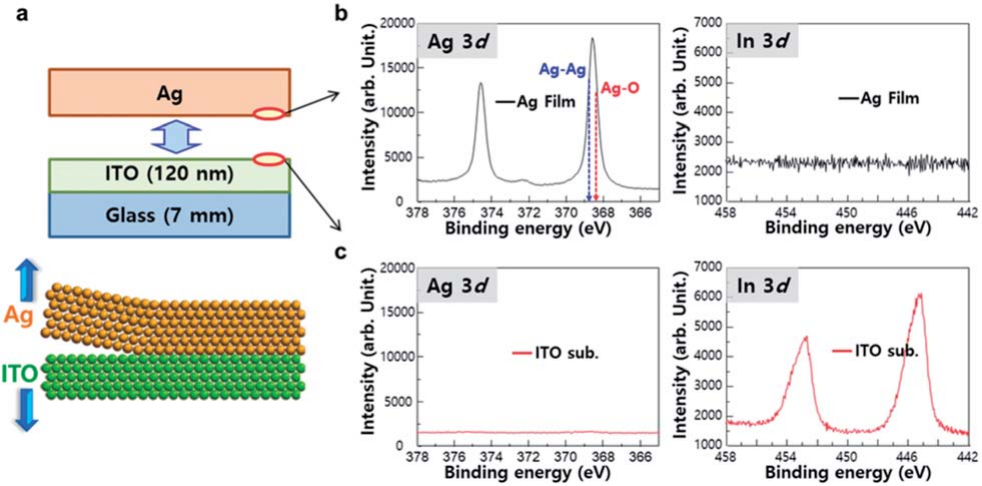
Interfacial reactions between Ag layer and ITO substrate on peel-off process. (a) Schematic drawing of Ag film peeled-off from ITO substrate. (b) Ag 3d and In 3d SRPES spectra of the surface of peeled-off Ag film. (c) Ag 3d and In 3d SRPES spectra at the surface of peeled-off ITO layer.

Chemical composition of surfaces of peeled-off film was *in situ* characterized in ultra-high vacuum using synchrotron radiation photoemission spectroscopy (SRPES). The Ag film coated on 120 nm-thick ITO-coated glass was peeled off (Fig. 2a) in the vacuum chamber, and Ag 3d and In 3d SRPES spectra were collected from both surfaces of peeled-off Ag film (Fig. 2b) and ITO substrate (Fig. 2c). The peak intensity (3d_5/2_) at 368.8 eV (Ag–Ag metallic bond) was stronger than the peak at 368.3 eV (Ag–O covalent bond), meaning that oxygen atoms on ITO have hardly bonded with Ag.^28,29^ There was no peak corresponding to In on the surface of Ag film. Similarly, no peak attributable to Ag was found on the surface of ITO substrate. This provides an evidence that there was no chemical reactions between Ag and the ITO interface. This lack of chemical reaction could be explained by the Gibbs free energy change to form an oxide, which is more negative for In_2_O_3_ (−831 kJ mol^−1^) and SnO_2_ (−520 kJ mol^−1^) than for AgO (−11 kJ mol^−1^).^30^

**Fig. 3:**
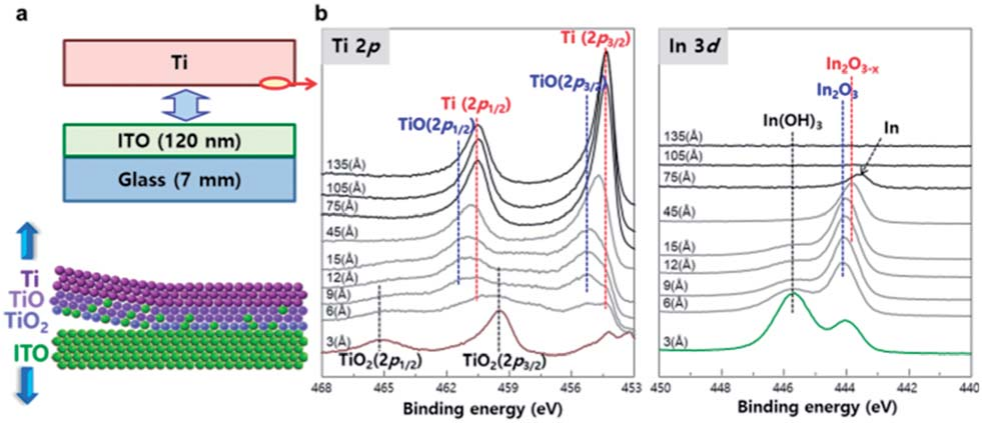
Interfacial reactions of Ti/ITO interface and depth profiling of Ti layer. (a) Schematic representation of peel-off reaction at Ti/ITO interface. (b) Ti 2p (left) and In 3d (right) spectra of lower side of peeled-off Ti layer as a function of sputtering depth.

To compare the peel-off mechanism between the reacting metal and non-reacting metal, the Ti layer on the ITO glass was peeled off (Fig. 3a), and the change in atomic composition with sputtering depth was quantified in the peeled-off Ti layer (Fig. 3b). At the Ti surface (3 Å sputtered), the peak (2p_3/2_) centered at 459.5 eV corresponds to the TiO_2_ fully-oxidized state that has a d^0^ configuration in the ground state.^31,32^ The peak at 455.3 eV could be assigned to the covalent TiO state.^33^ These peaks are related to the atomic oxygen of oxide-like structures characterized by covalent Ti–O bonding. As the depth of the sputtered Ti layer reached 45 Å, a sharp metallic peak appeared. The intensity of the metallic Ti peak increased with sputtering depth. These results suggest that the surface of the Ti layer had been oxidized by the ITO layer. The depth profile of the In 3d region demonstrates that metallic In^0^ compounds from the ITO layer were transferred to the peeled-off Ti layer. The chemical composition of In–O bonds changed with sputtering depth; *i.e.*, from highly-oxidized In(OH)_3_ to In_2_O_3_ to In_2_O_3−*x*_ to metallic In,^33^ then the signal of metallic In disappeared at sputtering depth ∼100 Å. The peeled ITO did not show signs of Ti (Fig. S3†). These results show that metallic Ti atoms react with In–O compounds of mother substrate and the In–O compounds diffuse into Ti metal layer. Unlike conventional adhesive interface that forms the inter-diffusion layer, the roughness of mother substrate can be transferred to the metal surface without leaving residues on both sides due to the stress caused by the plating layer formed on the top.

**Fig. 4:**
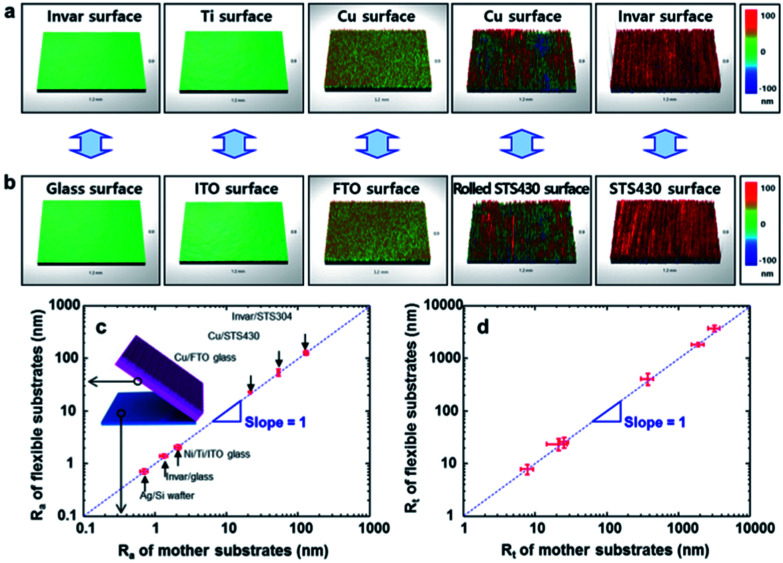
Surface roughness transfer from mother substrates to flexible metal films. (a) Peeled-off metal surfaces, and (b) peeled-off mother substrates for invar/glass, Ti/ITO, Cu/FTO, Cu/Rolled STS430 and invar/STS430 interfaces, (c) average roughness (*R*_a_) of flexible metal films *versus R*_a_ of mother substrates and (d) maximum roughness amplitude (*R*_t_) of flexible metal films *versus R*_t_ of mother substrates.

The transfer of roughness from the mother substrate to metal film was examined with the various type of substrate. AFM and 3D-profiler were employed to measure the surface roughness of flexible metal film (Fig. 4a) and mother substrates (Fig. 4b). Several kinds of substrates such as ITO glass, fluorine-doped tin oxide (FTO) glass, and STS430 steel, were used as a mother substrate, and invar, Ti and Cu were used as the flexible metal film to clarify the relationship between the surface roughness of flexible metal films and that of mother substrate. Si wafer (*R*_a_ = 0.7 nm), glass (*R*_a_ = 1.3 nm) and ITO-coated glass (*R*_a_ = 2.1 nm) were smoother than conventional steel substrates such as rolled STS430 (*R*_a_ = 53.0 nm; mechanically polished) and STS430 (*R*_a_ = 129.9 nm). The surface roughness of metal films depends on the roughness of mother substrate. That is, roughness was measured in sequence Ag@Si < invar@glass < Ti/Ni@ITO < Cu@FTO < Cu@rolled STS430 < invar@STS304 (Table 1), and the roughness was evaluated within 2% from their mother substrates (Fig. 4c and d).

Average (*n* = 5) surface roughness of peeled-off metal film (top 3 values in column) and mother substrate (bottom 3 values in column)

**Table:** 

Metal film	Ag _(AFM)_	Invar	Ti	Cu	Cu	Invar
*R*_a_ (nm)	0.7	1.4	2.1	22.9	54.1	127.2
*R*_q_ (nm)	0.9	1.8	2.6	28.8	71.1	180.4
*R*_t_ (nm)	7.9	23.6	25.7	406.2	1830.0	3724.3

**Table:** 

Mother substrate	Si _(AFM)_	Glass	ITO	FTO	Rolled STS430	STS430
*R*_a_ (nm)	0.7	1.3	2.1	21.5	53.0	129.9
*R*_q_ (nm)	0.9	1.7	2.6	27.2	70.5	173.2
*R*_t_ (nm)	7.8	21.2	25.3	374.6	1895.7	3180.0

This result indicates that the roughness of mother substrate was exactly transferred to the detached surface of the flexible metal films. This is the first result that EF metal films with *R*_a_ < 1 nm could be obtained using this surface roughness transfer method without any polishing or planarization process.

**Fig. 5:**
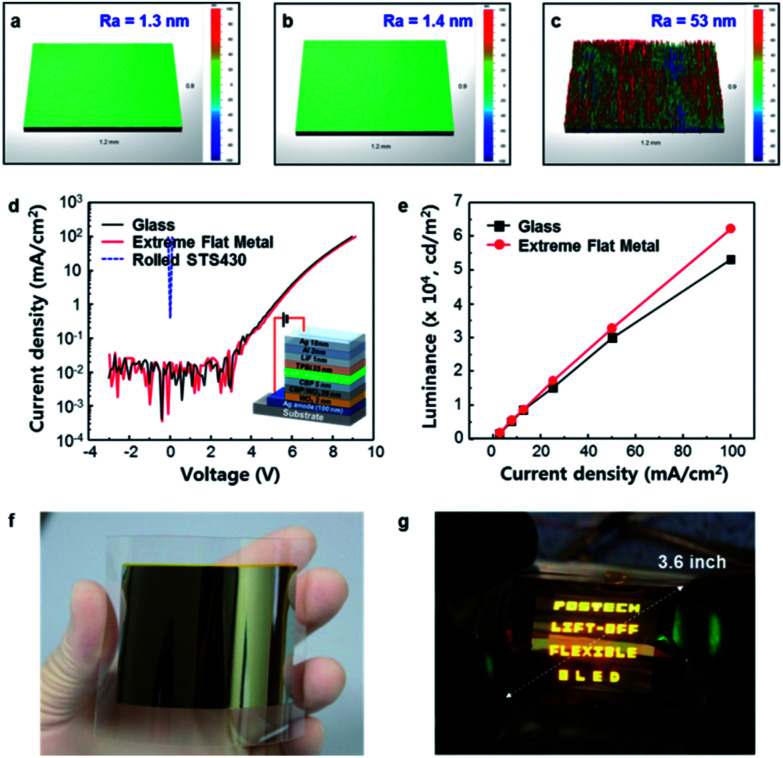
OLEDs' characteristics with surface roughness. (a) Surface roughness of glass (*R*_a_ = 1.3 nm), (b) EF Cu (*R*_a_ = 1.4 nm), and (c) rolled STS430 (*R*_a_ = 53.0 nm) using a 3D profiler. The measured substrate area was 1.2 mm × 0.9 mm. (d) Current density–voltage and (e) luminance–current density characteristics of OLEDs according to the type of substrate. (f) Optical image of a 3.6-inch EF metal film was fabricated by the surface roughness transfer technology. (g) The 3.6-inch OLEDs with the EF Cu substrate (Movie S1†).

In order to evaluate the effects of surface roughness on the performance of optoelectronic devices, top-emitting organic light-emitting diodes (OLEDs; CBP : Irppy_3_, *λ* = 510 nm) were fabricated on various substrates with different surface roughness, such as glass (*R*_a_ = 1.3 nm), EF Cu (*R*_a_ = 1.4 nm) and rolled ST340 (*R*_a_ = 53.0 nm) (Fig. 5a–c). The current density-operating voltage (*J*–*V*_OP_) characteristics of OLEDs showed that the device on glass and EF Cu had very similar current density (*J* = 25 mA cm^−2^) at *V*_OP_ ≈ 7.5 V; neither device showed leakage current (Fig. 5d). In contrast, the OLED fabricated on the rolled STS 430 substrate did not work due to leakage current, originated from a high surface roughness (*R*_a_ = 53 nm, *R*_t_ = 1.9 μm). The OLED on EF Cu had generally higher luminance *L* at a given *J* than that on glass (Fig. 5e). At *J* = 25 mA cm^−2^, the OLED with EF Cu substrate had *L* = 17 100 cd m^−2^, whereas the device on glass had *L* = 15 142 cd m^−2^. This could be due to the better heat dissipation from the OLED on EF Cu substrate than that on glass one (Fig. S4†). To confirm the applicability of the surface roughness transfer method to large area substrate, OLEDs were fabricated on a 3.6-inch EF Cu substrate (*R*_a_ = 2 nm) (Fig. 5f). All devices emitted light without defective cells when bent in N_2_-filled glove box (Fig. 5g). Also, the EF Cu was evaluated as a flexible substrate in organic photovoltaics (OPVs). It was found that electrical properties of OPVs on EF Cu substrate were comparable to those on glass one (Fig. S5†).

## Conclusions

In summary, we fabricated large-area EF metal films by peeling-off the metal film from the mother substrate. To compare peeling-off behaviors depending on the type of metal, surface components of non-reactive Ag and reactive Ti were analyzed after they were peeled-off from ITO mother substrate. The interfacial reaction, investigated by *in situ* synchrotron XPS, evidenced that diffusion of the substrate components at the Ti/ITO interface rather than the Ag/ITO interface and the different peeling-off behaviors between them. Despite variations in the materials and methods of forming the metal film, the detached metal film had surface roughness that was within 2% of that of the mother substrate. Because the EF metal film from roughness transfer method has low surface roughness, excellent thermal conductivity and thermal stability, flexible OLEDs and OPVs fabricated on EF metal films showed superior performance compared to devices fabricated on rigid glass substrates. The novel peel-off approach to fabricate EF film without any additional process, so it would be very useful for mass production of flexible, low-cost, high-throughput and high-smoothness substrate for flexible electronic devices.

## Conflicts of interest

There are no conflicts to declare.

## Supplementary Material

RA-008-C8RA00298C-s001

RA-008-C8RA00298C-s002
