# Supplementary material for: Extremely flat metal films implemented by surface roughness transfer for flexible electronics
Source: RSC Adv. 2018 Mar 19;8(20):10883–8. doi: 10.1039/c8ra00298c (PMC9078984; doi:10.1039/c8ra00298c)
Supplement: RA-008-C8RA00298C-s002 [file RA-008-C8RA00298C-s002.pdf]

Electronic Supplementary Material (ESI) for RSC Advances.
This journal is © The Royal Society of Chemistry 2018

Supporting Information

Extremely flat metal films implemented by surface roughness transfer for flexible electronics

Kisoo Kim,‡[a] Sungjoo Kim,‡[a] Gwan Ho Jung,[a] Ilhwan Lee,[a] Sungjun Kim,[a] Juyoung Ham,[a] Wan Jae Dong,[a] Kihyon Hong,[a,b] and Jong-Lam Lee[a]*

[a.] Division of Advanced Materials Science and Department of Materials Science and Engineering, Pohang University of Science and Technology (POSTECH), Pohang, 790-784 (Korea).

[b.] Present address: Department of Materials Science and Engineering, Chungnam National University, Daejeon, 34134 (Korea)

* E-mail: jllee@postech.ac.kr

Supplementary figures, Fig. S1-S5

Movie S1

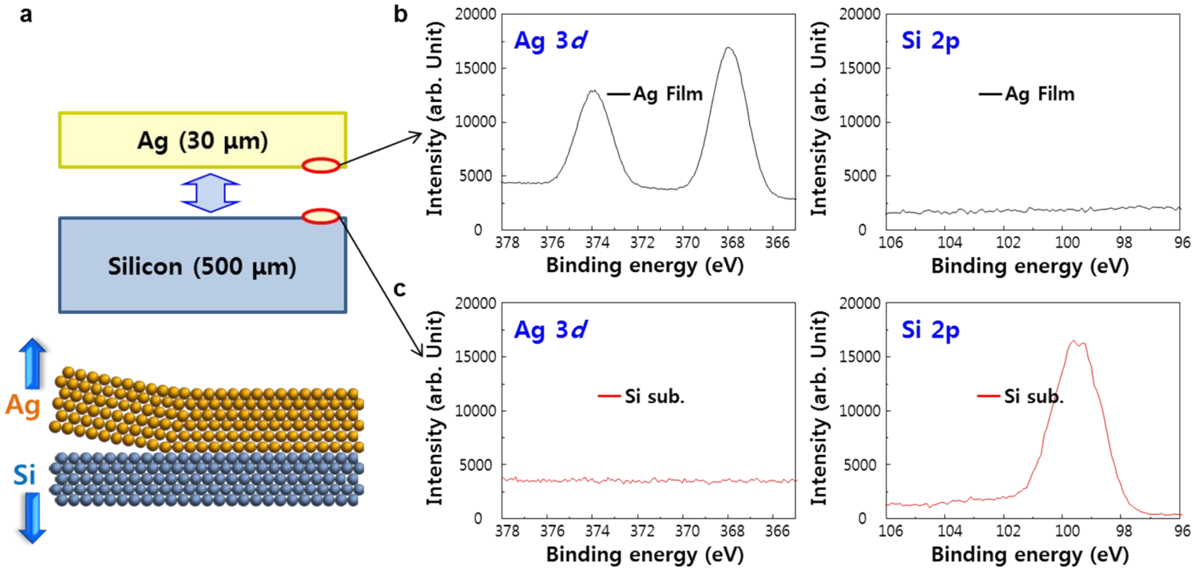

Fig. S1. (a) Schematic representation of peel-off reaction of Ag/Si interface. (b) Ag 3d and Si 2p XPS spectra of peeled off Ag layer, (c) Ag 3d and Si 2p XPS spectra of peeled Si wafer.

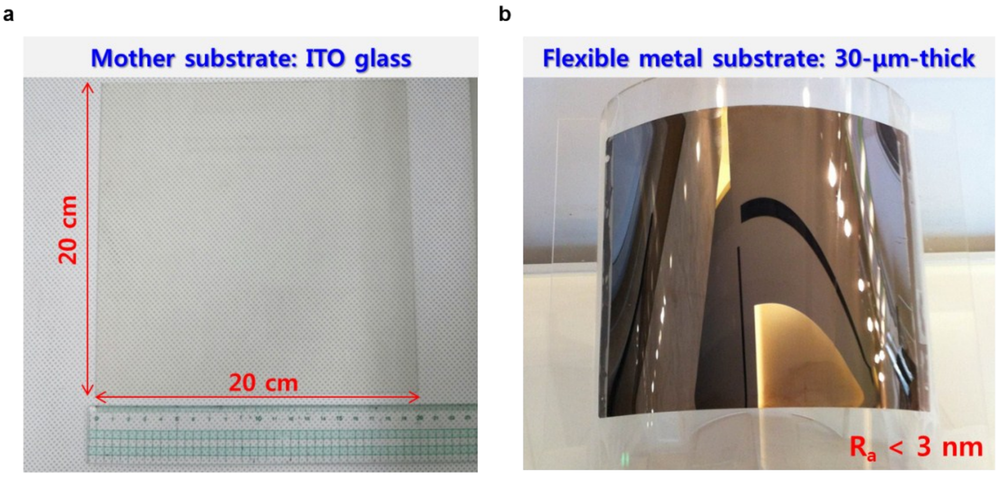

Fig. S2. (a) Mother substrate: ITO glass (20 cm × 20 cm size), (b) Peeled-off large area Cu flexible metal film (30-µm-thickness).

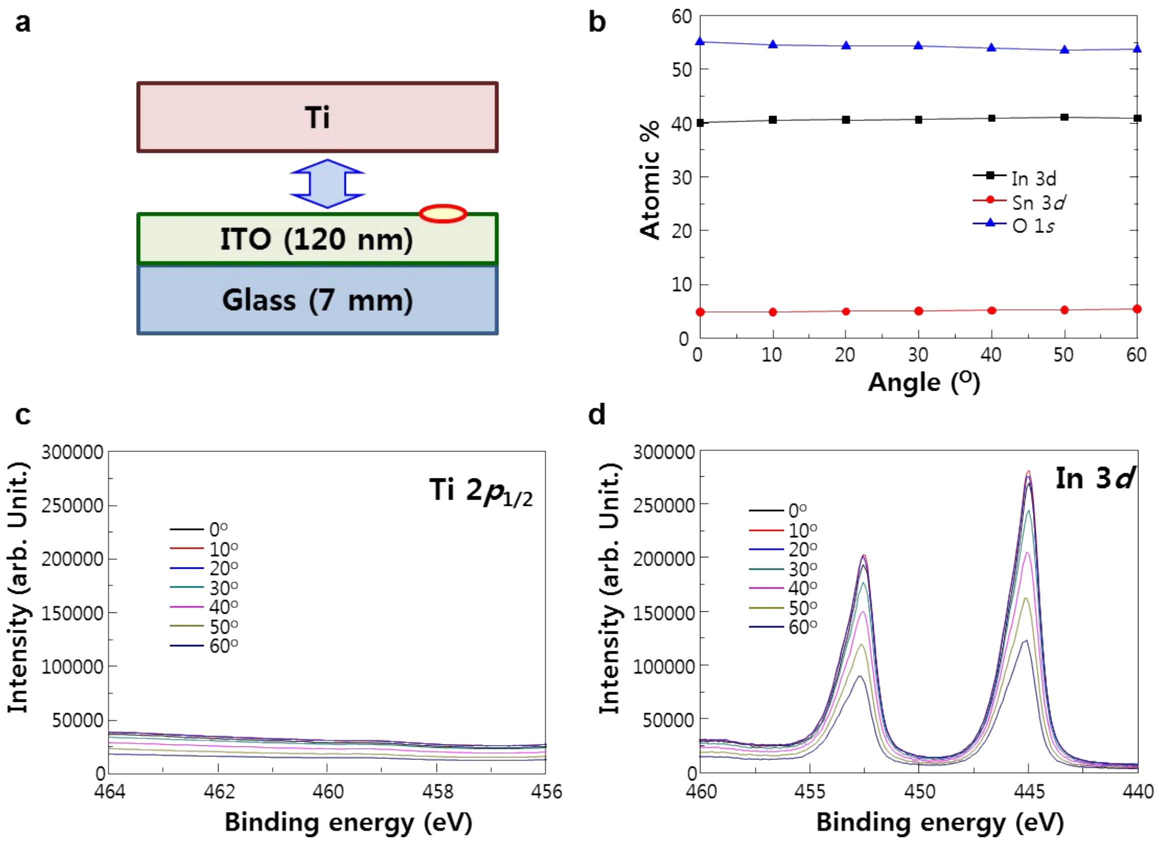

Fig. S3. Analysis of component change of mother substrate after peel-off with Ti layer using an angle-resolved photoemission spectroscopy. (a) Schematic representation of the peel-off process. (b) Atomic percentage in ITO layer as function of detecting angle. (c) Ti 2p (d) In 3d spectra in ITO layer after peel-off process.

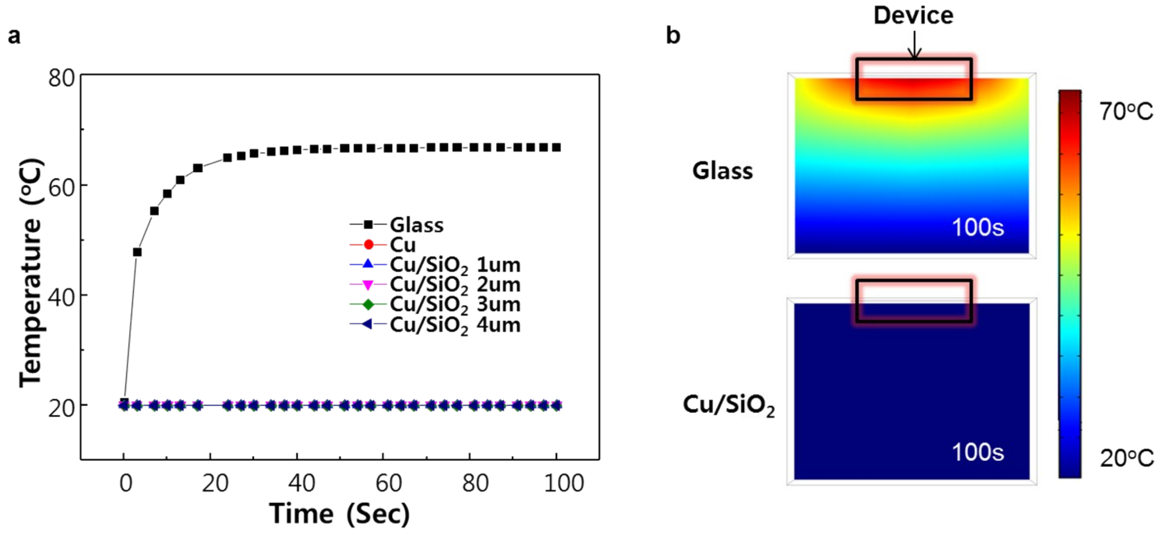

Fig. S4. ABAQUS simulation result of thermal degradation induced OLED device operation. (a) Temperature change between glass and metal substrate as function of time, and (b) Images of temperature distribution profile after operating 100 sec.

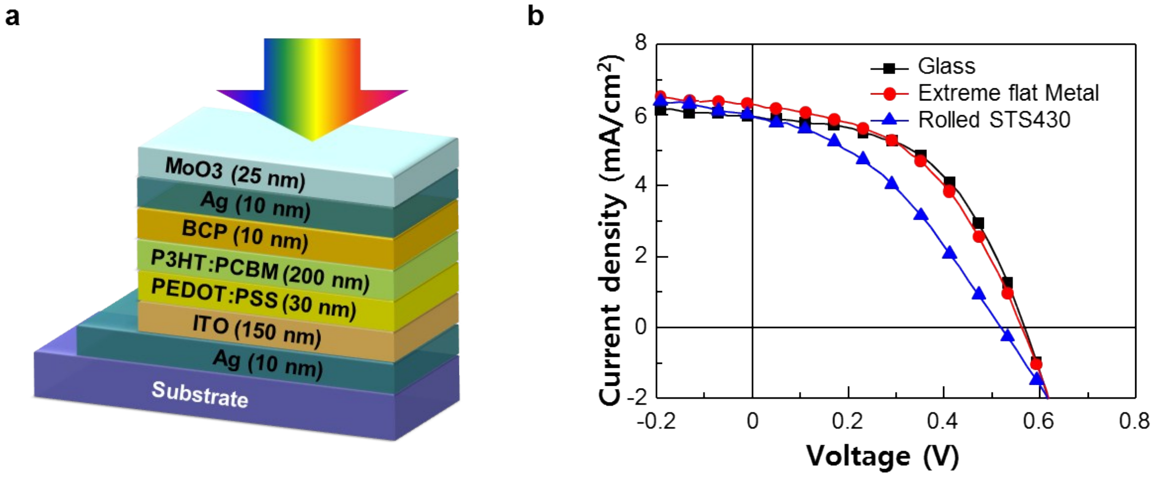

Fig. S5. (a) Schematic device structure of polymer solar cells. (b) Current density-voltage curves of polymer solar cells according to type of substrate.

Current density-voltage curves were obtained from devices with Glass, *EF* Cu, and rolled STS430 substrate (Fig. S5). The standard device was illuminated from the BCP/Ag/MoO_3 cathode. The standard device with the glass substrate (R_a = 1.33 nm, R_t = 21.19 nm) as a reference sample exhibits open circuit voltage V_{OC} = 0.57 V, short circuit current J_{SC} = 5.95 mA/cm^2, fill factor (FF) = 51.1 %, and power conversion efficiency (PCE) = 1.7 %. In the device with the *EF* invar substrate (R_a = 1.40 nm, R_t = 23.62 nm), the electrical characteristics (V_{OC} = 0.56 V, J_{SC} = 6.31 mA/cm^2, FF = 46.7 %, and PCE = 1.7 %) were comparable to those in the standard one. In contrast, devices fabricated on the rolled STS430 (R_a = 53.0 nm, R_t = 1.89 μm) consistently V_{OC} and FF responses. In the plot, V_{OC} = 0.52 V, J_{SC} = 5.16 mA/cm^2, FF = 38.3 %, and PCE = 1.0 % for a device fabricated on the rolled STS430. The device characteristics of glass and *EF* Cu samples were within the margin of experimental error due to similar *EF* surface roughness, whereas the electrical characteristics of the OPV in the rolled STS430 sample were poor due to its rough surface.

Movie S1. Movie demonstrating the OLEDs with the EF Cu substrate.
